# Supplementary material for: The interactome of intact mitochondria by cross-linking mass spectrometry provides evidence for coexisting respiratory supercomplexes
Source: Mol Cell Proteomics. 2017 Dec 8;17(2):216–32. doi: 10.1074/mcp.RA117.000470 (PMC5795388; doi:10.1074/mcp.RA117.000470)
Supplement: Supplemental Data [file supp_RA117.000470_133922_0_supp_23978_fzffwf.zip › spectra_annotation/mito_spectra_annotation/156-1-4-1-3-1.pdf]

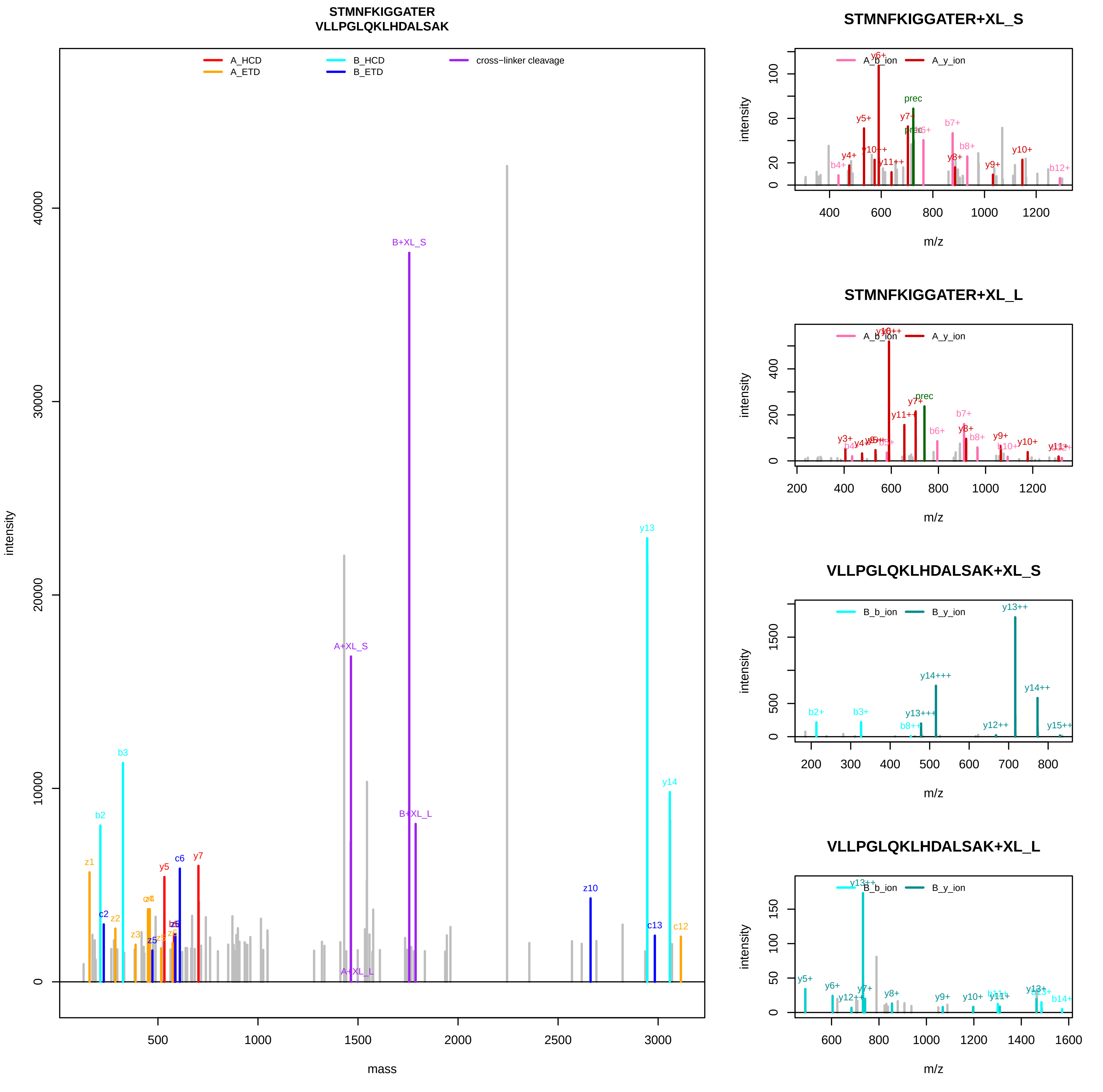

STMNFKIGGATER
VLLPGLQKLHDALSAK
A_HCD
A_ETD
B_HCD
B_ETD
cross−linker cleavage
intensity
mass
B+XL_S
A+XL_S
B+XL_L
A+XL_L
y13
y14
b3
b2
z1
c2
z2
z3
c4
z5
y5
c6
y7
z10
c13
c12
STMNFKIGGATER+XL_S
A_b_ion
A_y_ion
prec
m/z
STMNFKIGGATER+XL_L
VLLPGLQKLHDALSAK+XL_S
B_b_ion
B_y_ion
y13++
y14+++
y14++
y12++
y15++
b2+
b3+
VLLPGLQKLHDALSAK+XL_L
